# Real time circular tagging is possible through radial acquisition

**DOI:** 10.1186/1532-429X-18-S1-P315

**Published:** 2016-01-27

**Authors:** Shokoufeh Golshani, Abbas Nasiraei-Moghaddam

**Affiliations:** Amirkabir University of Technology, Tehran, Iran (the Islamic Republic of)

## Background

Real-time capturing of the myocardial function through CMR tagging is of great clinical importance. In circular tagging, tagline information manifests itself in k-space in an annular sub-region. This k-space characteristic in combination with polar acquisition schemes can be exploited to speed up the imaging. In our previous works, we showed that by selectively acquiring only this portion of k-space through circular sampling, it is feasible to capture tagline information with an acceleration factor of 8 [1]. We further described a coherent acquisition/reconstruction approach for radial tagging based on radial sampling of k-space [2]. In this study, we investigated the feasibility of that approach for circular tagging and evaluated its performance on phantom as well as human tagged images acquired with various numbers of radial projections.

## Methods

MR images with circular tag pattern were acquired from phantom as well as a healthy volunteer through a radial k-space sampling scheme on a 1.5T Siemens TIM Avanto scanner. The phantom data was collected with 504 radial spokes to fulfill the Nyquist sampling rate for a 192192 image matrix which prescribes acquisition of 302 projections. The in-vivo data, acquired with 88 views in 11 heartbeats, consists of 19 frames of a mid-ventricular short axis slice. A Hankel-based algorithm referred to as Polar Fourier Transform (PFT) is implemented for reconstruction of the acquired polar data. To study the performance of the method on undersampled datasets, we subsampled the raw datasets to a much lower extent than the Nyquist rate varying from 252 to 18.

## Results

**Figure 1:**
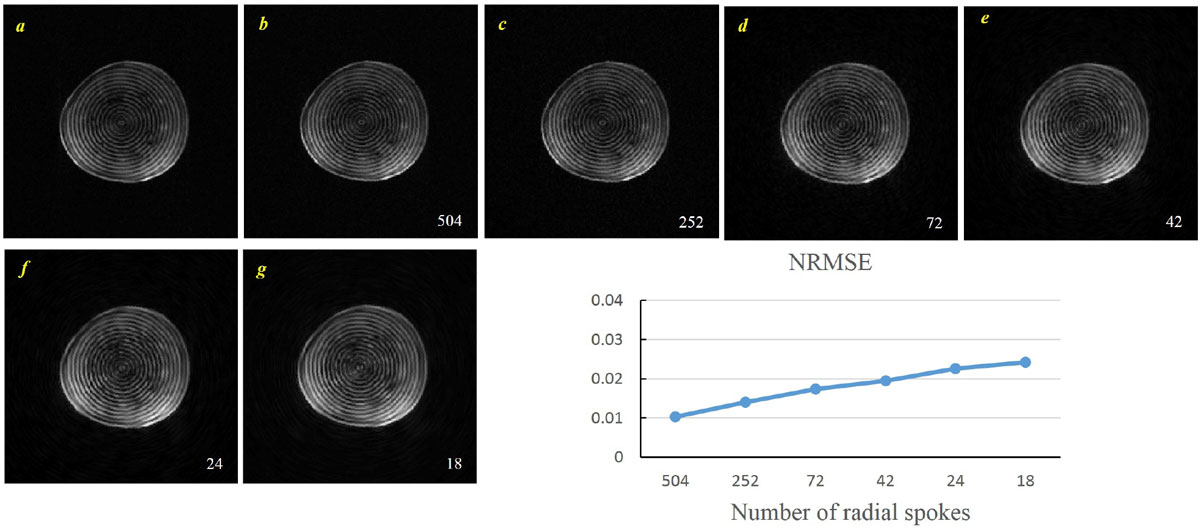
**Reconstruction performance and robustness of the proposed PFT method**. (a) Reconstructed phantom image by the MR scanner obtained with 504 radial spokes and (b) the corresponding image reconstructed by the PFT method. (c-f) undersampled images reconstructed through the PFT method. The right panel illustrates the Normalized RMSE for the images in terms of the acceleration factor. It is clearly seen that the proposed PFT method renders images without any visible streaking artifacts and the quality of taglines have been preserved. Only some blurring effects occurred in the periphery rather than the region of taglines.

**Figure 2:**
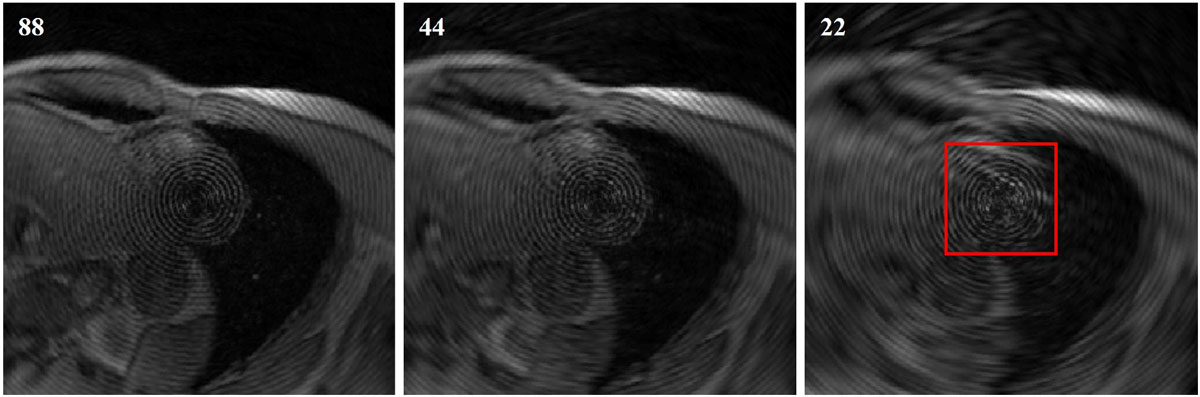
**(In-vivo images) Reconstructions of in-vivo images using our adapted PFT method for 88, 44, and 22 spokes**. The quality of taglines within the myocardium region (red square) has been greatly retained. The less the number of radial spokes, the shorter the scan time, however, this speed comes at the expense of losing some image quality.

Phantom data are shown in Figure 1. Panel (a) was reconstructed by MR scanner using the available re-gridding algorithms for radial readout. Panels b-g demonstrates reconstructed images from the original data (504 radial spokes) and undersampled images obtained by the adapted PFT method. The Normalized RMS Error for each reconstructed image is depicted in terms of the number of spokes in Figure 1 (bottom-right panel). Figure 2 (a-c) illustrates the in-vivo images of the first cardiac frame with original 88 radial views and the corresponding undersampled images with 44 and 22 views, respectively. The artifacts within the 22-view image are tolerable (NRMSE = 0.0310 relative to the 88-view image). It should be noted that the 22-spoke is close to the real-time imaging conditions [3].

## Conclusions

The PFT method in combination with radial k-space sampling is highly consistent and robust for circular tagging enabling undersampling factors of 16 that can be exploited towards real-time cardiac imaging. In contrast to commonly used reconstruction techniques for radial acquisitions, which su?er from severe streaking artifacts with undersampled projections, the overall appearance of PFT images even for extreme undersampling is of diagnostic quality.
